# The meaning of dignified care: an exploration of health and social care professionals’ perspectives working with older people

**DOI:** 10.1186/1756-0500-7-854

**Published:** 2014-11-27

**Authors:** Deborah Kinnear, Veronika Williams, Christina Victor

**Affiliations:** Dundee Dental Hospital and School, University of Dundee, Dundee, UK; Nuffield Department of Primary Care Health Sciences, University of Oxford, Gibson Building, 1st Floor, Radcliffe Observatory Quarter, Woodstock Road, Oxford, OX2 6GG UK; College of Health and Life Sciences, Brunel University, London, UK

**Keywords:** Dignity, Health care professionals, Social care professionals, Older people, Ageing, Care, Hands on care

## Abstract

**Background:**

Despite well established national and local policies championing the need to provide dignity in care for older people, there continues to be a wealth of empirical evidence documenting how we are failing to deliver this. While we have evidence as to what older people and their relatives understand by the term ‘dignified care’ we have less insight into the perspectives of staff regarding their understanding of this key policy objective. This paper aimed to explore the meaning of dignified care from the perspective of health and social care professionals’ working with older people. In-depth interviews and focus groups with health and social care professionals were carried out across four NHS Trusts in England, as part of a larger study, to investigate how dignified care for older people is understood and delivered. A total of 48 health professionals took part in in-depth interviews and 33 health and social care professionals participated in one of eight focus groups.

**Results:**

Health and social care professionals defined the meaning of dignified care as: ‘*dignity is the backbone of care’*, *‘it’s the “little things”’*, *‘feeling safe and secure’*, *‘treat as you want to be treated’, ‘treat as an individual’ and ‘Dignity encompasses multiple factors’*. ‘Hands on’ aspects of care were rarely mentioned when defining dignity. This suggests that policies around providing dignified care are being interpreted as an approach towards care and not with direct care provision. This limited interpretation of dignity may be one factor contributing to the continued neglect of older people in acute settings.

**Conclusions:**

These findings highlight that proactive measures are required to ensure that both relational and ‘hands on’ aspects of care are met for all older people receiving care in NHS trusts.

## Background

The care that older people receive has been the focus of intense public concern across the United Kingdom (UK) over recent years. There is a growing body of evidence suggesting that dignified care of older people being cared for is still compromised [1–13]. In February 2013 Robert Francis, Inquiry Chairman, released a report into the serious failings at the Mid Staffordshire NHS Foundation Trust, which included numerous instances of appalling care of older patients [4]. The report highlights that between 2005 and 2008 the Trust failed to tackle a dangerous negative culture involving an acceptance of poor standards and a disengagement from managerial and leadership responsibilities. Scandals such as the Mid Staffordshire report unfortunately are not uncommon. The Parliamentary and Health Service Ombudsman report [7], for example, details 10 cases of older patients who died after being admitted to NHS hospitals but who did not receive the most basic standards of care such that they were left without food or water, were soaked in urine or lying in faeces and left on the floor after falling. The NHS Operating framework for 2012–2013 [14] prioritises the care of older people stating ‘*some parts of the NHS are failing to provide elderly and vulnerable patients with dignified and compassionate care or to offer good standards in areas such as nutrition*, *continence and communication*’ (p.2).

While we know much about dignity, it remains a complex concept, subject to a range of different interpretations [15]. A philosophical model of dignity and its relevance to older people has been advanced by Nordenfelt [16] and his comprehensive analysis divides dignity into four types including: merit, moral status, personal identity, and universal human dignity (referred to by the German word Menschenwürde).

The first three types of dignity (merit, moral status and personal identity) are subjective and depend upon external influences. Dignity of merit refers to an individual’s role and status in society in which others recognise and respect the person for his or her accomplishments or position. Dignity of moral stature refers to a sense of self respect based on a personal sense of integrity in living one’s life. Dignity of identity is of the most relevance to discussion of dignity and ageing. This kind of dignity can be taken away from individuals by external events, by the acts of other people as well as by illness, injury and old age [16]. Nordenfelt [16] defines it as ‘*the dignity that we attach to ourselves as integrated and autonomous persons, persons with a history and persons with a future with all our relationships to other human beings’* (16, p. 75). Menschenwürde or universal human dignity, is completely different in that it refers to a kind of dignity that as humans we all have just because we are humans [16]. Thus, no individual can be treated with less respect than anybody else with regard to basic human rights. For example, an older patient in hospital should be treated in the same way as younger patients as they have the same basic human rights.

Maintaining their dignity while in hospital is of paramount importance to older people [11] and treating an individual with dignified care may positively influence both their treatment and social outcomes [17]. There is a wealth of research focusing on the older patient’s perspective [17–22] and the family carers’ perspective [23–26] citing respect, privacy, communication and being treated as an individual as important aspects of dignified care. However, they also emphasize the fundamental and vital aspects of care such as eating, nutrition, personal hygiene and toileting [11, 17–22]. Limited in the research literature is the professional perspective on the meaning of dignity and delivering dignity in care and, more specifically, the educational, cultural and organisational factors which enable or hinder its delivery. This is an important oversight as it is the attitudes, skills and behaviour of frontline staff via the development of organisational culture, policies and practice which is critical to the tangible delivery of policy imperatives [27].

Ariño-Blasco, Tadd and Boix-Ferrer [28] carried out one of the few studies exploring the professional perspective and more specifically a qualitative study with 85 focus groups which involved a total of 424 professionals in six European countries to determine health and social care professionals’ views of various aspects of dignity and older people. Participants were purposefully selected to represent different occupational groups, different levels of experience and seniority and the provision of care in different settings. Participants included medical, nursing, managerial, paramedical and social work professions from a range of settings, including hospital, residential and community. A total of 55 (13%) men and 369 (87%) women took part with a mean age of 41.42 years and their views of what constitutes dignified care were highly consistent: dignified care promotes autonomy, independence, engenders respect, maintains individual identity, encourages involvement, adopts effective communication practices and is person-centred and holistic. Similarly, Baillie [29] also carried out a qualitative study (case study design) investigating patient dignity in acute hospital settings. Both patients and ward-based staff took part in the study and they identified the following feelings as being central to the meaning of dignity: feeling comfortable, in control and valued, physical presentation and also behaviour. Patients expressed feeling comfortable if they felt safe, happy, relaxed, not worried, did not feel embarrassed and had a sense of wellbeing. A more recent study was carried out by Hall and Høy [30] exploring the professional perspective and more specifically, 29 Danish nurses’ experiences of caring for older hospital patients. Helping patients regain their dignity was considered to be of central importance to nurses and they reported that dignity was a value that had to do with integrity, respect and worthiness; something the older patients were at risk of losing when being hospitalised. Cairns et al. [31] also carried out a recent survey investigating the meaning and importance of dignified care from a health and social care professional perspective. A total of 192 professionals described the meaning of dignified care in terms of their relationships with patients including: ‘respect’ (47%), ‘being treated as an individual’ (40%), ‘being involved in decision making’ (26%) and ‘privacy’ (24%). ‘Being treated as an individual’ and ‘maintaining privacy’ were ranked as the most important components of dignified care while the physical caring tasks such as ‘helping with washing, dressing and feeding’ were rarely described as being part of dignified care and attributed much less importance than the relational components.

While there have been a few studies addressing the challenges faced by health care professionals in delivering quality care to older people [5, 27–32], very few sought to explore professionals’ understanding, conceptualization or definitions of dignity. If dignified care for older people is to be implemented successfully, we need to fully understand not only the patient and family carers’ perspective but also the professionals understanding of dignity in order to develop appropriate and relevant policies and procedures to avoid the breaches of dignity in care of older people.

This paper reports findings on a key research question from in-depth interviews and focus groups of health and social care professionals which forms part of a larger case study (survey, interviews and focus groups) exploring how dignified care for older people is understood and delivered by health and social care professionals and; how organisational structures and policies can promote and facilitate, or hinder, the delivery of dignified care. The purpose of the interviews and focus groups was to explore further the survey findings which have been reported elsewhere [31] and understand why direct ‘hands on’ aspects of care are accorded less importance in comparison to relational aspects of dignified care. In order to do so, we first had to understand the meaning of dignified care from a professional perspective before addressing why these were considered important. The research question that forms the specific focus of this paper is as follows:-

What does dignified care mean for health and social care professionals?

## Methods

### Data collection

Ethical approval for the study was obtained (REC ref number: 10/H0711/49) from both Brunel University and the UK National Research Ethics Service (NRES). Participants for focus groups and interviews were recruited from 4 different NHS sites. Health and social care professionals received information leaflets and reply slips through their ward/ clinical area administrators or managers and were asked to respond to the research team directly should they be interested in either taking part in a focus group or individual interview, or both.

#### Focus groups

Focus groups were arranged on-site (i.e. within the hospital/Trust premises) in a private and convenient place to participants (meeting rooms, seminar rooms or staff rooms). Two researchers were present for each focus group, one to lead the discussion and one to observe and take field notes. Prior to commencing the discussion, the researchers (DK and VW or WM) introduced themselves, explained the tape recording and reiterated that the discussion would remain anonymous. Once consent was received from all participants, the tape recording commenced and one of the researchers started the discussion using an interview guide.

#### Interviews

Interviews were conducted as one-to-one in depth interviews in a private room within the site premises or at the participant’s home if requested. Once consent was given, the interview commenced using an interview guide. All interviews were audio-recorded with the exception of two participants who requested that the researcher (DK) write notes instead. Both of these interviews were particularly sensitive in that the interviewee had experienced particularly poor practice examples and/or had poor working relations with their colleagues.

The purpose of the focus groups was to understand the professional (group) perspectives of dignity in care for older people and the organisational context of each site (i.e. acute, community, mental health). The purpose of the interviews was to explore individual’s experience and perspectives of dignity in care, thus enhancing the large quantitative survey [31] and focus group data.

### Data management and analysis

#### Focus groups & interviews

The qualitative data from the focus groups and interviews together augmented the quantitative contextual information collected as part of the larger project, which has been described elsewhere [31] providing more detail about the individual and organisational management of dignity within the range of settings. The qualitative data were managed using NVivo10 and analysed thematically. Audio recordings (focus groups and interviews) were reviewed and coded to determine key themes emerging from these data in relation to our research questions (for example, what does dignified care mean to you?). The process of analysis was ongoing and consisted of being immersed in the data and reading through it several times. A bottom-up approach was then used to see which categories and themes arose naturally from the data, rather than having prescribed categories and trying to fit data into these. The process of coding was initially carried out by the research fellow (DK). A sub-set of the interviews and focus groups were also analysed by a second researcher (VW). Finally, all data analysis was discussed and verified/challenged at regular team analytical meetings, thus ensuring group validation of the emerging themes. Each theme or category was verified by searching through the data for comparisons and challenges so that the themes could be refined and all the data accounted for.

## Results

### Profile of participants

We anticipated carrying out a total of 13 focus groups and 50 interviews in order to obtain a sufficient breadth and depth of information from across the four trusts. During the process of data collection and analysis we felt that, after 8 focus groups and 48 in-depth interviews, no new information was emerging and therefore saturation had been reached and no further primary data was required.

#### Focus groups

A total of 33 health and social care professionals participated in one of eight focus groups. Focus groups consisted of between 3 and 6 participants. The majority of participants were female (n =30); participants age ranged from 19 – 60 (mean age 40) years and job roles included: nursing role (n =16), occupational therapist (n =7) and mental health practitioner (n =4). The remaining 6 participants included roles included one each of a diverse range of roles (e.g. psychiatrist, psychologist, physiotherapist and social worker). Two of these participants included roles within a mental health trust that are unique and therefore not mentioned to ensure participant anonymity (See Table 1 for full participant profile).

**Table 1:** **Interview and Focus Group Demographic Information**

Demographic information	Interviews	Focus groups
*Gender*	*n*	*n*
Males	3	3
Females	45	30
*Total*	*48*	*33*
*Age*	*n*	*n*
Mean age	41 (range from 20 - 56)	40 (range from 19-60)
*Job Role*	*n*	*n*
Nursing Background	16	16
Physiotherapist	1	1
Psychiatrist	1	1
Occupational Therapy	19	7
Mental Health Practitioner	0	4
Manager	4	0
Health Care Assistant	1	0
Social Worker	0	1
Radiographer	1	0
Psychologist	1	1
Other	4	2
*Trust*	*n*	*n*
Trust 1	7	3
Trust 2	7	6
Trust 3	5	0
Trust 4	29	24

#### Interviews

A total of 48 health care professionals took part in in-depth interviews. The majority of participants were female (n =45) and their age ranged from 20 – 56 (mean age 41) years. Participant job roles included: nursing role (n =16), occupational therapy role (OTs) (n =19) and managers (n =5). The remaining 8 participants included one each of a diverse range of roles (e.g. clinical psychologist, radiographer and psychiatrist). Four of these participants included roles within their trust that are unique and again not mentioned to ensure patient anonymity (See Table 1 for full participant profile). Five participants took part in both an individual interview and in a focus group.

Data collection was carried out between June 2012 and November 2012.

### The meaning of dignified care

**Figure 1:**
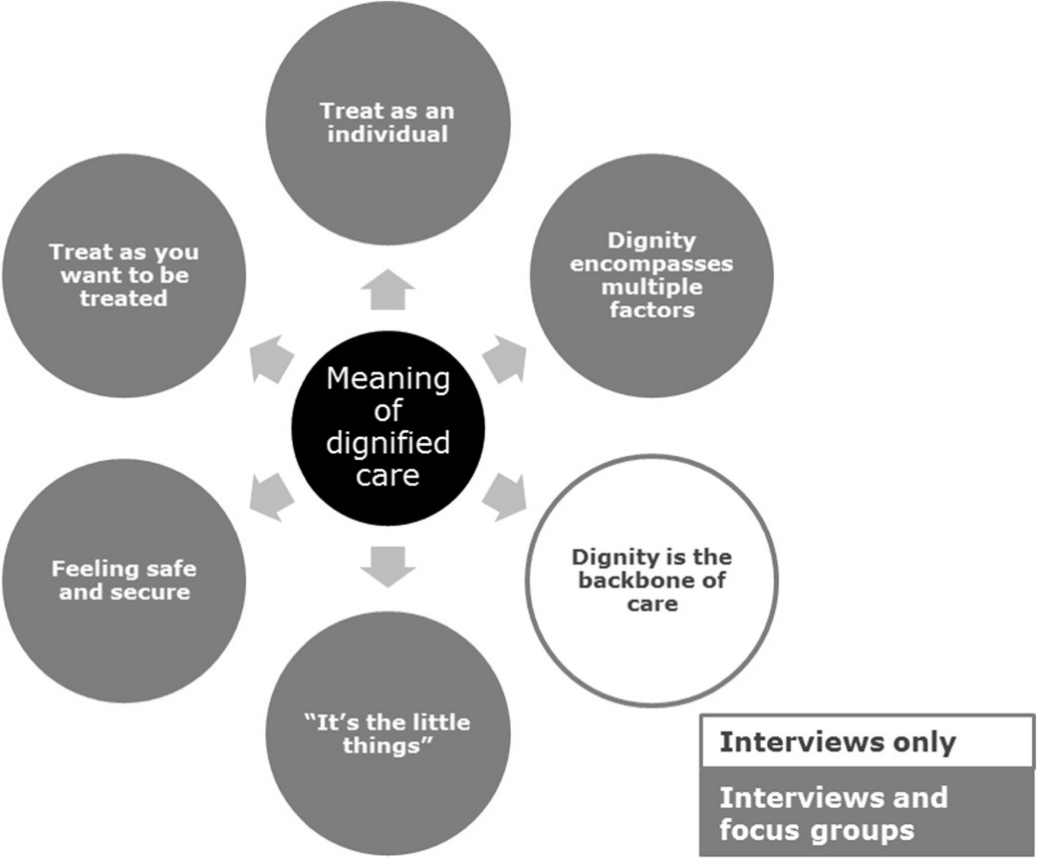
**The Meaning of Dignified Care (48 interviews and 8 focus groups).**

In both the interviews and focus groups participants were asked to describe in as much detail as possible and in their own words what dignified care meant to them. The following diagram (see Figure 1) summarises the main themes identified from participant responses.

#### Dignity is the backbone of care

A number of participants, all from a nursing background, described the central role that dignity played within their profession:

*“I think it [dignity] underpins my own values as a nurse. It’s what I, when I came into nursing I think it’s what I understood nursing was all about, and that hasn’t really changed throughout the whole of my nursing career and I think it’s what, it’s what I would want as a patient and it’s what I would expect to deliver as a nurse. It’s part of my code of conduct. It’s part of me”* (Interview 14)

*“it [dignity] is the basis of nursing. Everything we do should be underpinned by dignified care, washing, dressing, communicating, listening, caring and understanding. If there is no dignity then what’s the point”* (Interview 19)

Thus, dignity was seen as underpinning the role of a nurse. One nurse consultant, who had been in her post for 17 years, described how dignity had always been considered the backbone of nursing and nurse training even though the word ‘dignity’ per se was not used:

This was emphasised further by a nurse manager:

*“it [dignity] was very much the, the backbone of nursing and nurse training. When you were giving somebody a wash you wouldn’t have stripped them off and just washed them. You would have put, you know, blankets or towels discretely over bits of their body. So it was very much part of it. But I don’t think we actually kept using the words. It was very much about how you are with a patient, they are in a very vulnerable state. This is what you need to do for them. So yes, it was very much part of it”* (Interview 25)

*“I think for a lot of us, certainly at a senior nursing level, we’ve felt that actually you shouldn’t need to have something separate because it’s probably, it’s intertwined into everything that we’re doing really. So that when we’re thinking about nutrition, dignity comes into it. When we’re thinking about pressure sore prevention, dignity comes into that. When we’re thinking about record keeping, dignity I would say, comes into that”.* (Interview 14)

These quotes therefore illustrate the central role which dignity plays in the delivery of care. Dignity was described not only as the ‘backbone of care’ but also as ‘the little things’ that professionals’ carried out in their day to day role.

#### Dignity is ‘the little things’

Communicating with patients in an appropriate and polite manner such as asking how he/she is feeling or ensuring that patients body parts are not being unnecessarily exposed was an example of dignity being ‘the little things’:

*“I feel sometimes when we talk about dignity we look at the big things but I think it’s addressing people, addressing your patients like “good morning Mr Smith, how are you today?” or “can I help you with something?”. To me that is treating the patient with dignity. I don’t know if that is too simplistic…just to not acknowledge them”* (Interview 5)

*"I just think I’d like to be involved in decisions or involved in my care, as opposed to having my care given to me I want to be part of it. You know, I’d like, you know, the small things, like what would you like to wear or, you know, if I didn’t fancy doing something today then I don’t want to do it today"* (Focus Group 7B)

These examples of dignity relate to the relational aspects of care, such as acknowledging patients and involving them in the decision making process, as opposed to direct ‘hands on’ aspects of care such as assisting patients with feeding or going to the toilet. Health and social care staff also provided more specific examples of dignified care which will now be discussed in the remaining sub-headings.

#### Feeling safe and secure

Health and social care staff were very conscious of the fact that patients were coming into an unfamiliar environment and part of their role was to ensure that they felt safe and secure during their stay, thus ensuring dignified care:

*“And not making them feel, making them feel secure I think is always a good one, personally”* (Interview 28)

*"A lot of our work is around discharge planning and that is around safety and acceptable risk. Before I worked with some people who were falling over, and you can’t stop people falling but it’s all around minimizing the risk and keeping them as safe as possible"* (Focus Group 8A)

Thus, staff felt that the environment, which is again a relational aspect of care, was a key aspect of the meaning of dignified care. If patients felt safe and secure in the environment in which they were being cared for, then they were being treated with dignity. In addition to feeling safe and secure, health and social care staff frequently discussed the importance of treating patients the same way as they would want to be treated or how they would treat a family member.

#### Treat as you want to be treated

One student nurse, who was on placement, described how she would not do anything to patients that she herself would find unacceptable:

*“…just to make sure then that you do things to other people which you accept yourself”* (Interview 16)

A similar example was provided by an occupational therapy assistant:

*“Basically in very, very simple terms it would be just treating anyone how I would expect to be treated or how I would expect and want a family member to be treated*” (Interview 24)

It was not uncommon for participants to describe providing a standard of care to patients that they themselves would expect or want for a family member. While health and social care staff highlighted the importance of treating patients as they would want to be treated, a number of staff also stressed that what they wanted was not necessarily what their patient would want. This is elaborated on in the next theme.

#### Treat as an individual

Addressing the individual needs of patients was considered as one of the main themes that captured the meaning of dignified care:

*"to treat them as a person, as an individual, but because they’ve come into hospital it doesn’t automatically change them as a person, they’re just someone whose ill, you know, not to stereotype people"* (Focus Group 4C)

To emphasise why individualised care was so important, Sarah, a nurse consultant described the care given to a patient who was dying:

*“…a lady who was dying a few weeks ago, and her carers, every day, sounds really silly, they bought her a packet of chocolate buttons, because she loved dairy milk and she wasn’t eating, but every day they would just give her these buttons that would melt on her tongue and they were buying that out of their own pocket”* (Interview 18)

Similarly, Julie, a student nurse, highlighted the importance of catering towards patients’ individual needs:

*"Keeping in mind what the emotions of the patient could be. And you cater towards that. For example, they might really love a shower really early in the morning say. So if that’s really, really important to them, then you know, you want to try and do that for them"* (Focus Group 1E)

Thus, a simple kind gesture, considering the patient’s individual needs, encapsulated the meaning of dignity.

#### Dignity encompasses’ multiple factors

While specific examples of the meaning of dignified care were provided by participants, many often highlighted that the meaning could not be described as one single concept but instead encompassed multiple factors:

*“Ensuring that patients are treated in an appropriate manner, that their every needs are met in the most dignified way possible. That they are respected, heard and listened to, that their privacy remains intact and that they feel that they are valued as an individual”* (Interview 6)

*“…it’s treating a person with respect…and obviously dignity can be in the physical sense and …what about in terms of sort of mental and emotional response. So there are sort of different aspects to it”* (Interview 35)

*"I’ve got the same as the others, privacy, consideration of patients, communication"* (Focus Group 7A)

One participant, after providing examples of what dignified care meant to her, drew attention to the fact that the meaning of dignified care was in fact difficult to define:

*“To me it is making sure that the patient feels valued, that they’re respected. And it’s a very nebulous thing isn’t it dignity?”* (Interview 10)

It is perhaps then unsurprising why so many different definitions of the meaning of dignified care were provided and clear ambiguities are apparent in relation to the meaning of dignified care from a professional perspective.

## Discussion

Our paper presents the perspectives of health and social care professionals on the meaning of dignified care. *‘Dignity is the backbone of care’* , *‘It’s the “little things”’* , *‘Feeling safe and secure’* , *‘Treat as you want to be treated’* , *‘Treat as an individual’* and ‘*Dignity encompasses multiple factors’* were the most frequently cited definitions and these closely resonate with dignity guidelines, protocols and definitions that are embedded in national/local policies and which mesh with the expressed views of older people [5, 19–22]. Both the interview and focus group analysis highlighted that *‘Dignity is the backbone of care’*. Within this theme participants described dignity as “underpinning” their values as a health care professional and being part of their “code of conduct”. It was within this theme that the more ‘hands on’ aspects of care or ‘basics in care’ were touched on by a few participants in relation to washing and nutrition. However, it must be noted that these examples were the exception as opposed to the norm, as older people and various national reports have emphasised the importance of direct ‘hands on’ aspects of care including eating, nutrition, personal hygiene and toileting as an important component of dignified care [18, 21]. *‘Dignity is the backbone of care’* is perhaps a theme that is unsurprising given dignity appears as a core value in professional codes and human rights declarations.

Participants also defined dignified care as *“the little things” .* Examples included: involving patients in the decision making process (e.g. asking them what they would like to wear); addressing patients in a respectful manner and; acknowledging patients. Again these examples relate to the relational aspects of care. As dignity was often described as a difficult ‘nebulous’ concept, it may be these ‘little things’ that translate into everyday care situations. The ‘big’ conceptual ideas, such as those defined by Nordenfelt, may not be possible to achieve at all times in a busy care environment with a high patient and staff turnover. Participants also considered patients *‘Feeling safe and secure’* as an aspect of dignified care. Ensuring patients felt warm, comfortable and had enough food and water, ensured that they were in a safe and secure environment. Again, while the ‘hands on’ aspects of care such as providing food and water were reported, this was only by two participants. Feeling comfortable and safe were also aspects of care that were central to patients and ward-based staff in a study by Baillie [29]. Both patients and ward-based staff identified the following feelings as being central: feeling comfortable, in control and valued, physical presentation and behavior [29]. Tadd et al. [33] reported that when patients were in an environment in which they did not feel safe or secure, their dignity was compromised.

In addition to *‘feeling safe and secure’* in the current study, participants frequently discussed the importance of treating patients the same way as they would want to be treated or how they would treat a family member. While participants highlighted the importance of treating patients as they would want to be treated, a number of staff also stressed that what they wanted was not necessarily what their patient would want. This is elaborated on in the final theme *‘treat as an individual’* whereby participants felt that treating patients as individuals and meeting their individual needs encapsulated the meaning of dignified care. Similar findings were reported in a qualitative study exploring the factors that contributed to stroke patients’ satisfaction with rehabilitation care following a stroke [34]. ‘To be treated with respect and dignity’ was found to be the core factor identified in interviews with older patients. The main factor was sub-divided into five subcategories: being treated with humanity; acknowledged as individuals; having their autonomy respected; having confidence and trust in professionals; dialogue and exchange of information [34].

Finally, *‘Dignity encompasses multiple factors’* captures the various examples that participants provided when defining the meaning of dignity including: respect; being treated as an individual; communication and; privacy. Similar findings were reported by Cairns et al. [31] where participants reported ‘*respect’ , ‘being treated as an individual*’ , ‘*being involved in decision making*’ and ‘*privacy*’ as the most frequently cited definitions. Although the term ‘dignity’ is embedded in many reports and papers, it is rarely defined and has been described as both vague [35] and elusive [34]. It is therefore unsurprising that multiple examples were provided to describe the meaning of dignified care by participants. The current study findings resonate with dignity guidelines, protocols and definitions that are embedded in national and local policies and which mesh with the expressed views of older people and family carers [5, 16, 19–24]. However, older people, family carers’ and various national reports also emphasize the importance of direct ‘hands on’ aspects of care including eating, nutrition, toileting and personal hygiene as an important component of dignified care [18, 21, 23–26]. However, from both the interviews and focus groups, a combined total of 81 participants, only 5 participants made any reference to ‘hands on’ care.

The staff in our study clearly conceptualised dignity as an approach to their role focusing upon ideas of respect, feeling safe and secure, individuality and patient involvement; findings that resonate with the survey findings from this study [31] and from previous studies looking at the professional perspective [24, 25, 29, 36]. However unlike patients, few of our participants considered the direct ‘hands on’ aspects of care provision such as feeding and toileting as defining dignified care. It is possible that the definitions described by professionals in this study are different from patient perspectives due to the way that policies and debates about dignity are portrayed. In the main they are concerned with attitudes about how care should be delivered rather than how care is delivered. Thus we hypothesize that policy makers and practitioners are ‘taking for granted’ the implicit delivery of care embedded within their roles and see dignity as being concerned with how care is delivered. It is also plausible to suggest that direct ‘hands on’ or fundamental aspects of care are being neglected in staff definitions of dignity because of the specialisation and separation of roles and the emphasis in policy documents on how care is delivered [33].

### Limitations

While the majority of participants in our study were female, this reflects the general NHS health care professional population [5]. With regards to the focus groups, we anticipated a minimum of 5 participants per group. Upon receiving confirmation of at least 5 participants, focus group dates and times were confirmed. However, due to the nature of the professionals being interviewed, a number of issues arose resulting in participants dropping out at the last minute. Reasons included staff shortages, emergencies on the ward and unexpected meetings. On instances where there were fewer than 5 people, participants were invited to be interviewed instead. On four occasions there were fewer than 5 people. However, all participants expressed the wish to go ahead with the focus group as planned. We therefore felt that is was important to continue with the focus groups to enable those who had attended the opportunity to discuss their experiences of dignified care. While our intention was to interview and conduct focus groups with both health and social care professionals, only one social worker took part in a focus group and no social care professionals came forward to be interviewed.

## Conclusion

Our study highlights the differences between staff, patient and family carers’ expectations as to what constitutes dignified care. Furthermore the lack of meaning attributed to the vital and fundamental aspects of ‘hands on’ care suggests that policies around providing dignified care are being interpreted as an approach towards care and not with direct care provision. As previously proposed [31] this limited interpretation of dignity may be one factor contributing to the continued neglect of older people in acute settings. Policy makers, NHS organisations, managers, medical doctors, nurses and health and social care professionals more generally, equally have a duty of care to address the vital aspects of dignified care. Thus, proactive measures are required in order to support and encourage health and social care professionals. One possible suggestion is ward leadership, where health and social care professionals are directed and supported in addressing and providing not only relational aspects of care but also direct ‘hands on care’ such as eating, nutrition, personal hygiene and toileting. Identifying the facilitators and barriers to delivering dignified care will add richness to these findings and help us better understand why direct ‘hands on’ aspects of care are considered with less importance [31].

### Ethics approval

Ethical approval for the study was obtained (REC ref number: 10/H0711/49) from both Brunel University and the UK National Research Ethics Service (NRES).

## References

[1] Department of Health (2001). National Service Framework for Older People.

[2] Department of Health (2001). The Essence of Care Patient-Focused Benchmarking for Health Care Practitioners.

[3] Charter of Fundamental Rights of the European Union (2000). Official Journal of the European Communities.

[4] Francis RF (2013). Report of the Mid Staffordshire NHS Foundation Trust Public Inquiry: Executive Summary. The Mid Staffordshire NHS Foundation Trust. Public Inquiry.

[5] Royal College of Nursing (2008). Defending Dignity – Challenges and Opportunities for Nursing, RCN London.

[6] Commission on Dignity in Care for Older People (2012). Delivering Dignity: Securing dignity in care for older people in hospitals and care homes. A report consultation.

[7] Parliamentary and Health Service Ombudsman (2011). Care and Compassion? Report of the Health Service Ombudsman on ten investigations into NHS care of older people. Ordered by the House of Commons.

[8] Care Quality Commission (2010). Inpatient survey.

[9] Commission A (2004). Older People – Independence and Well-being: the Challenge for Public Services.

[10] Help the Aged (2008). On our own Terms: The Challenge of Assessing Dignity in Care.

[11] Healthcare Commission (2006). Living Well in Later Life. A Review of Progress against the National Service Framework for Older People.

[12] Department of Health (2004). Better health in Old Age: A Report from Professor Ian Philp, National Director for Older People's Health to Secretary of State for Health.

[13] Care Quality Commission (2011). National Overview. Dignity and Nutrition Inspection Programme.

[14] Department of Health (2011). The Operating Framework for the NHS in England 2012–13.

[15] Tadd W, Gunning J, Holm S (2007). “Dignity and Older Europeans. Ethics, Law and Society. Vol. III.

[16] Nordenfelt L (2004). The varieties of dignity. Health Care Anal.

[17] Tadd W, Dieppe P, Bayer T (2002). Dignity in health care: reality or rhetoric. Rev Clin Gerontol.

[18] Older People’s Commissioner for Wales (2011). ‘Dignified Care?’ The Experiences of Older People in Hospital in Wales, Wales.

[19] The Patients Association (2011). ‘We’ve been listening, have you been learning?.

[20] The Patients Association (2010). Listen to Patients, Speak up for Change’.

[21] Woolhead G, Tadd W, Boix-Ferrer J (2006). ‘Tu’ or ‘Vous’? A European qualitative study of dignity and communication with older people in health and social care settings. Patient Educ Counsel.

[22] Calnan M, Badcott D, Woolhead G (2006). ‘Dignity under threat? A study of the experiences of older people in the United Kingdom’. Int J Health Serv.

[23] Jangland E, Gunningberg L, Carlsson M (2008). Patients’ and relatives’ complaints about encounters and communication in health care: evidence for quality improvement. Patient Educ Counsel.

[24] Wilkes L, White K, O’Riordan L (2000). Empowerment through information: supporting rural families of oncology patients in palliative care. Aust J Rural Health.

[25] Jamerson PA, Scheibmeir M, Bott MJ, Crighton F, Hinton RH, Kuckelman A (1996). The experiences of families with a relative in the intensive care unit. Heart Lung.

[26] Bridges J, Flatley M, Meyer J (2010). Older people’s and relatives’ experiences in acute care settings: Systematic review and synthesis of qualitative studies. Int J Nurs Stud.

[27] Maddock S (2002). Making modernization work: new narratives, change strategies and people management in the public sector. Int J Public Sector Manage.

[28] Arino-Blasco S, Tadd W, Boix-Ferrer JA (2005). Dignity and older Europeans: the voice of professionals. Qual Ageing Policy Pract Res.

[29] Baillie L (2009). Patient dignity in an acute hospital setting: a case study. Int J Nurs Stud.

[30] Hall EO, Hoy B (2012). Re-establishing dignity: nurses’ experiences of caring for older hospital patients. Scand J Caring Sci.

[31] Cairns D, Williams V, Victor C, Oliver D, Martin W, Richards S: **The meaning and importance of dignified care: findings from a survey of health & social care professionals.** *BMC Geriatr* 2013.,**13**(28)**:** doi:10.1186/1471–2318–13–28 10.1186/1471-2318-13-28 PMC3614439 23517491

[32] Ball EB, Murrells T, Rafferty AM, Morrow E, Griffiths P (2013). ‘Care left undone’ during nursing shifts: associations with workload and perceived quality of care. BMJ Qual Saf.

[33] Tadd W, Hillman A, Calnan S, Bayer T, Read S (2011). Dignity in Practice: An exploration of the care of older adults in acute NHS Trusts. Preventing Abuse and Neglect in Institutional Care of Older Adults.

[34] Pullman D (2004). Death, dignity and moral nonsense. J Palliatiave Care.

[35] Shotton L, Seedhouse D (1998). Practical dignity in caring. Nurs Ethics.

[36] Maben J, Adams M, Peccei R, Murrells T, Robert G (2012). ‘Poppets and parcels’: the links between staff experience of work and acutely ill older peoples’ experience of hospital care. Int J Older People Nurs.

